# A Capability Perspective on Antibiotic Resistance, Inequality, and Child Development

**DOI:** 10.1007/978-3-030-27874-8_14

**Published:** 2020-04-06

**Authors:** Michael Millar

**Affiliations:** 2 Monash Bioethics Centre, Monash University, Melbourne, VIC Australia; 3 Monash Bioethics Centre, Monash University, Melbourne, VIC Australia; Barts Health NHS Trust, London, UK

**Keywords:** Antibiotics, Capabilities and growth stunting, Social justice, Mother and child health, Infectious Disease

## Abstract

Nussbaum’s capability theory by drawing attention to multiple determinants of wellbeing provides a rich and relevant evaluative space for framing antibiotic resistance. I consider the implications of antibiotic resistance for child development and adult capabilities. There are common risk factors for childhood growth stunting and the spread of infectious diseases in both antibiotic sensitive and resistant forms. The interaction between infectious diseases, antibiotic resistance and growth stunting illustrates a clustering of disadvantage. The control of antibiotic resistance requires wide-ranging cooperative action. Cooperation is predicated on an expectation of equitable access to effective antibiotics. This expectation is confounded by inequality both in access to antibiotics, and in the risk that available antibiotics will be ineffective. Securing child development (and adult capabilities) requires that inequalities both in access to antibiotics and in risk factors for the dissemination and transmission of antibiotic resistance are addressed. Inequality undermines the cooperative activity that is control of infectious diseases and compounds the threat to the securing of capabilities that arises from antibiotic resistance.

## Introduction

 Antibiotic resistance has been framed as a problem consequent on the lack of development of new antibiotics and overuse of existing antibiotics. How we frame a problem is important in determining our responses to the problem (Tversky and Kahneman 1981). Unsurprisingly solutions to antibiotic resistance have been focused on developing new antibiotics, and constraining the use of existing antibiotics (see for example http://drive-ab.eu, and https://www.bu.edu/law/faculty-scholarship/carb-x). Yet, new antibiotics and constraints on use of existing antibiotics can never be a solution to the potentiation of infectious disease transmission (in antibiotic sensitive or resistant forms) consequent on poverty, over-crowding, malnutrition, limited educational opportunity, environmental degradation, poor water quality, inadequate sanitation or conflict.

## Capability Theory

Capability theory has been influential in defining measures of human development, and quality of life, and in the evaluation of the justice of social arrangements. Sen (1999) defined a capability as a ‘substantial freedom he or she enjoys to lead the kind of life he or she has reason to value’. Nussbaum emphasises the importance of capabilities for human dignity, and derives entitlements from reflecting on the requirements for equal dignity and respect. She describes necessary conditions for a decently just society, in the form of a set of ten fundamental entitlements for all citizens (Nussbaum 2006). More recently capability theory has been applied to children (Biggeri et al. 2011) and child development (Peleg 2013). Nussbaum and Dixon (2012) have proposed that capability theory can be used to provide theoretical justification, and to justify a degree of special state priority for children’s rights – based on the ‘unique vulnerability of children to the decisions of others’ (Nussbaum and Dixon 2012, p. 575).

 Antibiotic resistance has implications for child development, basic capabilities, and the securing of adult capabilities. There is on-going debate about the elements that should be included in a capabilities list that is appropriate for adults (Wolff and De-Shalit 2007) or children (Biggeri and Mehrotra 2011). Children develop in to adults, moving through different developmental stages and capabilities at different ages. Child development both depends on the capabilities of adult carers, and determines the potential for adult capabilities. For the purposes of this chapter I have accepted the list that Nussbaum proposes as appropriate for adults, and that achievement of thresholds of adult capabilities is substantially dependent on child development. The capabilities listed by Nussbaum (2006, p. 154) encompass life expectancy, bodily health & integrity, sense, imagination and thought, emotions, practical reason, affiliation, relations with other species, play and control over one’s environment.

## Infectious Disease and Capabilities

The use of antibiotics can be conceptualised as an attempt to try to prevent damage caused by infection to established capabilities (adults) or the potential for capabilities (children). One consequence of the use of antibiotics is antibiotic resistance. The rate at which antibiotic resistance develops is a function of usage, time, control measures, and the context of use. The availability of effective treatments for infectious disease is a substantial determinant of health. The interactions between health and the determinants of health are complex and not unidirectional, so for example health both determines and is determined by nutrition, education, and social status. The extent of capability fulfilment can be used to define health, while health is required for the fulfilment of capabilities (Venkatapuran 2011, 2013). Uncontrolled infectious disease subverts the achievement of adult capabilities through multiple pathways including through damage to child development. Damage to the capabilities of adult carers also has consequences for child development.

The control of infectious diseases (antibiotic sensitive and resistant) requires fulfilment of multiple entitlements. An entitlement to bodily health requires that an individual is able to have good health, including reproductive health; to be adequately nourished; and to have adequate shelter. The adequacy of shelter is important as a risk factor for the spread of infectious disease, and for damage to child development (see for example Shelter 2006). Nutrition influences both child development and infectious disease susceptibility (Gough et al. 2014). Maternal reproductive health is an important determinant of child development and vulnerability to infectious disease. A capability for senses, imagination, and thought requires education. To secure child development we must secure the capabilities of those who care for them. Nussbaum and Dixon (2012) emphasise that ‘the goal remains the full empowerment of all individuals’ (p. 578). Maternal education is a key element in assuring healthy child development (see discussion of growth stunting below). Maternal education and parental control of their local environment are necessary elements in protecting children from infectious disease. The entitlements to be able to play and to have relationships with the world of nature can be qualified by adding ‘safely’. Children in developing countries may not be able to live and play without exposure to the risk of infectious diseases (antibiotic sensitive and resistant) transmitted as a result of poor sanitation, close proximity to animals with zoonotic infection, and vectors for disease (such as malaria mosquitoes). Infectious disease contributes to impairment of child development through multiple pathways including growth stunting (discussed further below).

The capabilities listed by Nussbaum remind us of important dimensions of the individual experience of infectious disease (such as freedom of movement and engagement in social interactions). The entitlement to affiliation requires that an individual is able to live for and in relation to others, to recognize and show concern for other human beings, and to engage in various forms of social interaction. Bodily integrity requires that an individual can move freely from place to place. These last two entitlements can be breached by restrictions taken to control the spread of infectious disease (antibiotic sensitive or resistant), and by the social consequences of infectious disease, particularly when associated with treatment (antibiotic ) resistance (see for example Upshur et al. 2009).

Constraints on freedoms to prescribe, to purchase, to manufacture and formulate, to dispose, to pollute, and to use antibiotics for economic gain can all contribute to the control of antibiotic resistance. Nussbaum’s approach does not preclude limitations on freedoms with respect to the use of antibiotics. Nussbaum gives emphasis to the need to limit freedoms when those freedoms adversely impact on the central capabilities. She states that ‘no society that pursues equality or even an ample social minimum can avoid curtailing freedom in very many ways, and what it ought to say is those freedoms are not good, they are not part of a core group of entitlements required by the notion of social justice, and in many ways, indeed, they may subvert those core entitlements’ (Nussbaum 2011, p. 73). ‘In other words, all societies that pursue a reasonably just political conception have to evaluate human freedoms, saying that some are central and some trivial, some good and some actively bad, some deserving of special protection and others not’ (Nussbaum 2011, pp. 74–75). Framing the actions, constraints and precautions from a capability perspective also identifies limits to the precautions that we can take and gives priority to actions, which do not undermine capability entitlements. A policy that results in a substantial loss of a capability for some can be challenged from a capability perspective, even if there was an overall benefit. Stigmatisation, isolation, and segregation of individuals to prevent the spread of treatment resistant infection (such as leprosy historically) would not be consistent with a capability perspective while there remain feasible alternative courses of action. The non-availability of effective treatments resulting from antibiotic resistance restricts alternative courses of action. For much of the twentieth century women with leprosy were actively discouraged or prevented from having children. New-born babies of mothers with leprosy were taken from their parents at birth, because otherwise the child would also develop leprosy (see for example International Leprosy Association, History of Leprosy). Capabilities related to childbirth including the opportunities to have and to look after a child were removed. Leprosy has now been controlled to a large extent by the advent and availability of effective antibiotic treatments.

## Human Dignity and Infectious Disease

An emphasis on the importance of human dignity is a substantial element within Nussbaum’s capability theory. There is a lack of consensus on how best to define and measure human dignity (Ashcroft 2005). Dignity can be defined positively but also negatively as freedom from sources of humiliation (see Shultziner and Rabinovici 2011). Nussbaum, while acknowledging that dignity is a poorly defined concept, uses human dignity as a touchstone for the selection of capabilities. Nussbaum’s list of capabilities is a list of positive entitlements (capabilities) that are necessary for the living of a life with human dignity. Respect for the dignity of others is an important social and political value. Conditions must pertain that reflect that value and these include conditions that foster a sense of personal worth in each person.

There are strong associations between social status, self-esteem and health (Mann 1998; Marmot 2003; Chilton 2006). There are many ways in which dignity interacts with infectious diseases. There are common risk factors for violations of dignity and infectious diseases, for example lack of access to safe toilets contributes to loss of dignity, gender-based violence, and the transmission of infectious disease (see WHO factsheet 392). Inadequate shelter threatens dignity and increases infectious disease risks (Shelter 2006). Some infectious diseases, particularly those that are difficult to treat, such as antibiotic resistant tuberculosis, increase the risk that the dignity of adults will be violated (Upshur et al. 2009). Children with HIV or with a parent with HIV can suffer a substantial loss of self-esteem (Chi and Li 2014). An individual or group with an infectious disease may suffer from stigmatisation, social isolation, and a resultant loss of a sense of self-worth. Social consequences of infection for individuals, groups and institutions also include blame and shame (Sontag 1989). Acquisition and carriage of antibiotic resistant bacteria by individuals while undergoing healthcare can be associated with stigmatisation (Rump et al. 2017). Public health policies designed to support the control of antibiotic resistant bacteria can also lead to stigmatisation of individuals (Ploug et al. 2015). Violations of dignity and damage to self-esteem can increase infectious disease risk through changes in human behaviour. Low self-esteem is associated with sexual risk taking behaviour and sexually transmitted disease (see Byrnes et al. 1999; Ethier et al. 2006).

There are common risk factors for violations of dignity and infectious diseases, violations of dignity increase infectious disease risk, and infectious disease increases the risk that dignity will be violated.

## Clustering of Disadvantage: The Example of Growth Stunting

In discussing actions which lead to the destruction of capabilities Nussbaum states that ‘We can certainly agree that capability-destruction in children is a particularly grave matter and as such should be off-limits’. ‘Usually situations are not so grave, and thus in many such cases the approach has little to say, allowing matters to be settled through the political process’ (Nussbaum 2011, p. 27). Unfortunately capability-destruction is not unusual in many countries. It is estimated that more than 40% of children in lower and middle income countries are at risk of impaired development (Black et al. 2016). A period of particular risk to development is that between conception and 3 years of age. More than 25% of children <5 years of age globally have stunted growth (low height-for-age). Stunting is associated with long-term cognitive and physical impairment (Hair et al. 2015; Noble et al. 2015) and substantial economic consequences for individuals, communities and countries (Horton and Steckel 2013). The WHO Conceptual Framework for stunted growth (Stewart et al. 2013) identifies a range of community and societal contextual factors underlying the causes of stunting of growth of children. These factors include political arrangements, poverty, regulatory frameworks, healthcare systems, beliefs and norms, the status of women, access to safe foods, sanitation, population density, and natural and manmade disasters. Solutions require intervention in multiple sectors with specific emphasis given to the way in which resources are controlled and distributed through the political and economic system, food security, education (particularly of females), water quality, sanitation (and hygiene), ameliorating poverty and vulnerability, and access to healthcare (Casanovas et al. 2013). Nussbaum’s capability approach specifies a broad range of entitlements and in so doing is well placed to explicitly accommodate these multiple and complex requirements.

Infections are thought to contribute to the pathogenesis of growth stunting and infections are both more common and more serious in children with stunting. Recently it has been suggested that administration of antibiotics to populations of children could be used to prevent stunted growth (Gough et al. 2014). The proposal to use antibiotics as a population level intervention to mitigate the risk of stunting illustrates the tension between sustaining effective antibiotics while assuring access for those in pressing need. Population level antibiotic interventions have profound implications for present and future generations particularly when the target populations live under conditions of relative deprivation that facilitate the spread of agents of infection in sensitive and resistant forms. Common risk factors for stunting and for the spread of infectious diseases include overcrowding, poor education (particularly maternal), poor nutrition, inadequate sanitation, and poor water quality. Capability insufficiencies (such as poor shelter, threats to bodily health, lack of access to maternal education) contribute both to the transmission of infectious disease and to host susceptibility to disease, and potentially to the burden of antibiotic resistance. There is a clustering of disadvantage in that infectious disease amplifies other disadvantages consequent on capability insufficiencies.

The use of antibiotics in many developed economies has extended beyond the treatment and prevention of life-threatening human infections to include the mitigation of symptoms of self-limiting disease(s), animal husbandry, fish farming, and to allow us to extend the range of medical and surgical interventions including those with limited health benefits such as some forms of cosmetic surgery. In developed countries antibiotics have become a means to a variety of ends, with varying degrees of relationship with mitigation of harm to human capabilities. Despite this there is evidence that the use of antibiotics in developed countries has stabilised or fallen this century (van Boeckel et al. 2014). Most of the recent increase in use of antibiotics has been in rapidly developing countries including Brazil, Russia, India, China and South Africa (BRICS countries). Holland particularly has shown how it is possible for developed countries to have low levels of human antibiotic usage and low levels of antibiotic resistance associated with patients (http://www.ecdc.europa.eu/en/activities/surveillance/EARS-Net/Pages/Index.aspx). By contrast antibiotic resistance is more prevalent now in developing countries with high levels of deprivation than in developed countries (WHO Report 2014). This situation probably reflects the capacity of countries to expend resources on trying to assure socio-economic conditions (and capability entitlements) such as education, health, nutrition, sanitation, and housing which are significant determinants of the epidemiology of infectious disease. An oft-quoted example of this relationship is that of tuberculosis. The incidence of tuberculosis (TB) and the requirement to treat TB has declined dramatically over recent decades in developed countries and this decline has been attributed to improvements in socio-economic conditions (Comstock 2000). By contrast with developed countries such as Holland the context of use of antibiotics in low to middle income countries frequently involves heightened conditions for the spread of infectious diseases both in antibiotic sensitive and resistant forms.

 Antibiotic resistance considered from within a capability perspective draws attention to the importance of the social, political and economic context in determining infectious disease risks. Antibiotic resistance threatens to place an additional burden on communities where growth stunting and infection are already prevalent, because many of the risk factors for growth stunting also determine the risk that antibiotic resistance will spread. Capabilities may be incommensurable but capabilities still interact with each other in contributing to a state of wellbeing. In the real world these interactions may lead to clustering of disadvantage (Wolff and De-Shalit 2007). The interaction of infection with growth stunting in children illustrates the clustering of disadvantage and the importance of addressing the broad range of capability deficiencies. Otherwise preventable childhood stunting has the potential to persist alongside burgeoning levels of antibiotic resistance.

## Capability Thresholds and Inequality?

‘The basic claim of my account of social justice is this: respect for human dignity requires that citizens be placed above an ample threshold of capability in all ten of those areas’ (Nussbaum 2011, p. 36). In this section I ask if capability thresholds can be achieved while there remains avoidable and substantial inequality in access to effective antibiotics and in the determinants of infectious disease. Inequality can be and often is harmful to human wellbeing (Picket and Wilkinson 2010). Our sense of self-worth, our social status, our wellbeing and inequalities are intertwined (Marmot 2003, 2005). Thresholds of capability may not achievable while substantial inequalities remain. Wolff and De-Shalit (2007, p. 10) define a society of equals as one in which ‘disadvantages do not cluster, where there is no clear answer to the question of who is the worst off’. Certainly countries are not equal partners and neither are individuals within countries, when account is taken of the burdens of infectious disease – as exemplified by the contrasting patterns of child development and the clustering of disadvantage.

There is inequality in access to effective antibiotics (Laxminarayan et al. 2016). Pneumonia is still responsible for 1 in 5 deaths of children less than five years old in the world today. Ensuring access of children with pneumonia to antibiotics has been a major objective of the WHO and UNICEF over the last decade (WHO 2013). There is also inequality in the risk of acquisition of antibiotic resistant bacteria. There is increasing evidence of extensive environmental contamination with antibiotics and antibiotic resistant bacteria (Lubbert et al. 2017), particularly from antibiotic manufacturing plants, from use of antibiotics in meat production, from hospitals and urban conurbations (Berendonk et al. 2015). There is increasing use of antibiotics to support animal meat production even in countries with high levels of childhood growth stunting (see Centre for Science and Environment Report 2014). It is estimated that by 2030 the use of antibiotics in livestock production in the US and China will account for 40% of global antibiotic use (Van Boeckel et al. 2015). Antibiotic resistant bacteria, selected in humans and animals, are shared through environmental pollution with both those who can and those who cannot afford to eat meat. Often those with less or no access to antibiotics live in conditions, which promote the spread of antibiotic resistance. These differences are particularly strong in countries where some live in relative affluence in close proximity to slums. Those who are better off have access to the best medical advice, diagnostics for antibiotic resistance, and the latest treatments including antibiotics. When antibiotic resistant bacteria contaminate the environment of people with inadequate sanitation, poor education and with a heightened susceptibility to disease then spread is facilitated. Contamination of the Ganges provides a specific example. While the rich can afford cremation on the Ganges, and pilgrimage to the upper Ganges, the poor cannot. The water of the Ganges has become highly polluted with antibiotic resistant bacteria including a particularly worrying form of antibiotic resistance called New Delhi Metallo-β-lactamase (NDM-1) (Ahammad et al. 2014). Poor people use the water of the Ganges for recreation, washing, and drinking. Poor people with limited access to medical care or antibiotics are exposed to extreme forms of antibiotic resistance through day-to-day activities, with implications for the efficacy of antibiotics, their health and for the health of those around them. There is inequality in the risk of acquisition of antibiotic resistance as well as inequality in access to effective antibiotics (see Note 1).

## International Cooperation, Unequal Partners

Currently the majority of countries collaborate on the control of infectious diseases. One hundred and ninety-six countries have signed up to the International Health Regulations (2005) (developed after the SARS outbreak in 2003), which are designed to control the international spread of infectious disease. The control of epidemic diseases is included in the United Nations Rights Document A/6316 (1996). Over recent years antibiotic resistance has become a focus of international concern. The World Health Organisation (2015) has developed an action plan for antimicrobial resistance, which recognises the need for international collaboration (for example see Section 21 (4), Global Action Plan). There is also broad acceptance in the scientific literature (see for example Institute of Medicine 2010) that control of antibiotic resistance requires international cooperative action.

Unequal relationships between countries can subvert cooperation. For example Indonesia has had outbreaks of avian influenza, which have been associated with high mortality, yet withdrew from cooperation with the WHO Global Influenza Surveillance Network (GISN) in 2007. The position from the Indonesian perspective is described in a journal article as follows – ‘Indonesia believes that the world must work in unity against the H5N1 virus infection…. The work must be conducted side by side with mutual trust, transparency and equity as global citizens professionals, taking into consideration the elements of human dignity and solidarity.’ ‘The avian influenza case in Indonesia has demonstrated once again the unresolved imbalance between the affluent ‘high-tech’ countries and poor agriculture-based countries. Countries that are the hardest hit by a disease must also bear the burden of the cost of the vaccine, therapeutics and other products, while the monetary and non-monetary benefits of these products go to the manufacturers that are mostly in the industrialised countries’ (Sedyaningsih et al. 2008, p. 487). Indonesia and other resource poor countries were expected to participate in cooperating in the control of infectious disease, yet there was a belief that the benefits of cooperation were unequally distributed. Subsequently an agreement was reached which stipulated arrangements for more equitable cooperative arrangements (World Health Assembly 2011).

Another example of an unequal international relationship that relates more directly to the control of antibiotic resistance arises from the Kumarasamy et al. report in 2010 that New Delhi Metallo-β-lactamase-1 was widespread in India. The Indian government considered this name to be ‘unfair’ and stigmatising, and potentially undermined the burgeoning health tourist market in India (see Pandey 2010). The Editor of the Lancet subsequently described the use of this name as an error and apologized stating that the name had ‘unnecessarily stigmatised a single country and city’ (Sinhal 2011). This interaction has compromised research on the epidemiology of NDM-1 in India according to the lead author of the Lancet report (Tim Walsh), who named this form of resistance NDM-1. He stated that ‘We were banned from India and India had a massive clampdown on sending (biological) strains out’ (Sugden 2013). Health tourism is developing fast in India and the stigma associated with the potential acquisition of antibiotic resistant bacteria was characterised by Indian politicians as an international plot to undermine that development. Unfortunately NDM1 is now globally distributed (Berrazeg et al. 2014). Conflict between the Lancet and the Indian government over health policy has continued (Sinhal 2015).

This second story illustrates aspects of the epidemiology of antibiotic resistance. When social conditions are poor then antibiotics quickly become ineffective, antibiotic resistant bacteria can be rapidly spread around the world, and there is a relationship between the sustaining of the functions of antibiotics and other socio-economic ‘goods’ such as adequate shelter, and clean water. This example also illustrates a relationship between antibiotic resistance, and stigma, which can undermine cooperation in the control of antibiotic resistance. There is considerable inequality in the international influence of the biomedical press with a bias towards developed countries with a strong research base. Combinations of economic inequality, antibiotic prescribing practice norms, and publication practice have marked out India in a way that adds additional disadvantage in a competitive global market for medical tourism.

## A Relational Approach to Capability Inequality

Nussbaum argues that a contractualist account (such as that proposed by Scanlon 1998) based on individuals as moral equals ‘is a powerful intuitive way of capturing the idea that human beings are moral equals despite their widely differing circumstances in an unequal world’ (Nussbaum 2006, p. 272). Nussbaum states that ‘I employ the notion of reasonable rejection, or something very close to it, in articulating my account of political justification’ (Nussbaum 2011, p. 89). Her approach ‘is a partial account of specifically political entitlements’ (Nussbaum 2011, p. 96).

Scanlon provides an account of why and when we can reject both distributive and non-distributive inequalities (see Scanlon 2003, 2013) which can be applied to Nussbaum’s evaluative capability framework. Reasonable rejection and justification to others provide the substantial focus of Scanlon’s contractualist approach (Scanlon 1998) (for more discussion of Scanlon’s approach see Note 2). Interactions between individuals and groups determine the epidemiology of infectious diseases. Human relations directly or indirectly have a substantial role in the spread of infectious diseases. In addition many of the transactions that determine the use of antibiotics and the consequences of use involve individuals, institutions and nations in dialogue. Examples include healthcare workers (such as doctors) agreeing treatment plans with patients, or healthcare authorities and institutions agreeing antibiotic policies within nations, or nations agreeing approaches to international collaboration on the control of antibiotic resistance. Justification is a key element to these relational interactions and it seems intuitively attractive to start from an acknowledgement of the importance of justification in assuring the validity of principles and agreements. Another attractive feature of Scanlon’s approach is a concern with assuring the conditions for self-worth. This is significant both in relation to the epidemiology of infectious disease (as previously discussed) and in relation to a concern with assuring the conditions for a life with human dignity (a substantial concern for Nussbaum’s capability theory) (Fitzpatrick 2008).

For Scanlon everyone counts morally, regardless of race, gender, or where they live. The different reasons for rejecting inequalities are dependent ‘on the way that an inequality affects or arises from the relations between individuals’ (see Scanlon lecture – Why does inequality matter?). Reasons for rejecting inequalities ‘presuppose some form of relationship or interaction between unequal parties’ and are based on comparing the differences in the situations of individuals (Scanlon (2004) lecture – When does equality matter?). For Scanlon (2006) reasonable rejection does not depend on rejection of the distribution of goods, but rather it depends on assuring equal respect and fairness. Nussbaum’s capability theory provides an evaluative framework for the extent to which equal respect and fairness are achieved, without being itself a complete theory of distributive justice.

Scanlon argues that ‘relief of suffering, avoidance of stigmatising differences in status, prevention of domination by others, and the preservation of conditions of procedural fairness are basic and important moral values’ (Scanlon 2003, p. 218) that can give reason to reject inequalities. Inequalities can also be rejected when there are institutional obligations to provide certain benefits in an even-handed way, or ‘cases in which individuals, as participants in a cooperative endeavour, have at least a *prima facie* claim to an equal share of the goods which that endeavour produces’ (Scanlon 2013, p. 463). The control of antibiotic resistance is a cooperative exercise. Cooperation is predicated on an expectation of a share in access to effective antibiotics. Importantly the scope of these reasons for rejection of inequalities extends beyond national boundaries (for a fuller discussion see O’Neill 2013).

## Inequalities Subvert Capabilities

Inequalities can lead to substantial disadvantage, furthering inequalities in a competitive world, and cluster to give multiple disadvantages. Inequalities can result in stigmatising differences in status, both at the level of the individual and the state as shown by the example of NDM-1 antibiotic resistance described above. Inequalities in access to antibiotics coexist with inequalities in risk factors for infection, and risk factors for antibiotic resistance. These inequalities can and do contribute to social, political and economic disadvantage, as exemplified by the interactions between childhood growth stunting, infectious diseases and antibiotic access.

Wealth determines access to healthcare, medicines (including antibiotics) and healthy living conditions. The less wealthy in many countries have less access to high quality medicines, little access to medical advice, or diagnostic facilities, often live under conditions that may facilitate the spread of antibiotic resistant bacteria, may be exposed to resistant bacteria even before exposure to antibiotics, and are more at risk of infection, and of the serious consequences from infection. Securing child development requires control of infectious diseases in treatment sensitive and resistant forms. There is an unequal distribution of childhood burdens and benefits associated with antibiotics and antibiotic resistance both within and between countries. These inequalities contribute to avoidable suffering (preventable infectious disease), differences in status (growth stunting), and potentially domination of vulnerable children by others. Inequalities subvert the achievement of capability thresholds.

## Addressing Inequalities, Achieving Capability Thresholds

Control of antibiotic resistance is a cooperative enterprise with shared objectives and shared responsibilities. Countries cooperate in the control of infectious disease including antibiotic-resistant agents of infection. If we accept that all of the parties engaged in the control of antibiotic resistance should have an equal *prima facie* claim to access to effective antibiotics then when inequalities exist we can ask if those inequalities can be justified – ‘basic structures need to be justified to all who are asked to accept them’ (Scanlon lecture – Why Does Inequality Matter?) (see Note 3).

Developed countries have used antibiotics for much longer and in much larger quantities and for a wider range of reasons than most developing nations. India is subject to criticism for insufficient regulation of antibiotic prescribing. Yet, even by 2010, India used half the number of antibiotic units per person compared with the USA (Laxminarayan and Chaudhury 2016). Historically while new antibiotics were regularly coming to the market there was no commercial motivation to constrain antibiotic use because antibiotic resistance was a major justification for using the new antibiotic (s). The profits from antibiotic sales were largely accrued by companies based in the developed world. Marketing decisions were dominated by consideration of profit maximisation with a relatively low priority given to public health. The United Nations has accepted that developed nations should take up a greater part of the responsibility for the control of greenhouse gases than developing countries. ‘Common but Differentiated Responsibilities’ (UNFCCC 2015) are justified because there are differences in capacity to respond, different priorities, and differences in the historical contribution to the problem of greenhouse gas emissions between developed and developing countries. These differences also apply to antibiotic resistance.

In the World Health Assembly Global Action Plan for Antimicrobial Resistance (2015) Section 21. (3) **Access** states that ‘*The aim to preserve the ability to treat serious infections requires both equitable access to, and appropriate use of, existing and new antimicrobial medicines’.* Currently despite the high level of international concern there is no international agreement as to what constitutes ‘appropriate’ use of antibiotics. Equitable access is a long way away when account is taken of the lack of access to antibiotics for the treatment of life-threatening infection in many parts of the world while antibiotics are used extensively without human health benefit in many other parts of the world. I have previously argued that appropriate use is that which prevents some substantial risk of irretrievable harm in patients or their contacts, where a substantial risk is a level of risk which exceeds the range of risks of irretrievable harm that we tolerate in our day to day lives (Millar 2012). Use of antibiotics to support (non-human) animal growth promotion is inappropriate use. Use of antibiotics for animal growth promotion has been reported to be increasing in both developed and developing countries (Van Boeckel et al. 2015), including in areas where human growth stunting is particularly prevalent such as Sub-Saharan Africa. In developing countries people are more likely to live in close proximity to animals, so that the risks that antibiotic resistant bacteria will spread from animals, and that people will be exposed to antibiotics present in their environment, is increased compared with developed countries where the close proximity of farm animals with people is less common. It is strikingly inappropriate for many children to have limited access to antibiotics while antibiotics are being used as animal growth promoters. Developed countries should take a lead in limiting use of antibiotics as animal growth promoters.

The UN has given a substantial place to human dignity in the Sustainable Development Goals (UN 2014). These goals include improvements in education, housing, nutrition, water quality, and sanitation. The scope for international action on antibiotic resistance includes the amelioration of the conditions that potentiate the need for antibiotics and the spread of resistant forms of agents of infection. Improving living conditions (including housing), nutrition, education (for example with respect to risk factors for disease), and other determinants of human dignity such as female empowerment have been shown to reduce the risk of stunting, but these are also important factors in the control of infectious disease transmission and potentially antibiotic resistance. Achieving UN Development Goals (assuming that fairly and honestly set – see Hickel 2017) for 2030 would do much to mitigate the risk factors for both stunting and the transmission of agents of infection in both antibiotic sensitive and resistant forms.

## Conclusions

Nussbaum’s capability theory (Nussbaum 2006) provides a rich and relevant evaluative space for framing antibiotic resistance. Securing child development and adult capabilities requires that we address inequalities in access, regulate ‘appropriate prescribing’, and clarify responsibilities for addressing inequalities in risk factors for the dissemination and transmission of antibiotic resistance. Historical and current patterns of antibiotic use impose a burden of responsibility on developed countries to ensure that their own use is appropriate, and raise questions with respect to the responsibilities of developed countries to address risk factors for antibiotic resistant infection in developing countries. Antibiotics and antibiotic resistant bacteria pollute the environment (particularly when sanitation standards are poor) – compounding the inequality and disadvantage of those without equivalent access to effective antibiotics. Inequality undermines the cooperative activity that is control of infectious diseases and compounds the threat to the securing of child development and adult capabilities that comes from antibiotic resistance.

### Note 1

There is some empirical evidence that European countries with more income inequality have higher levels of antibiotic resistance than those with less inequality (Kirby and Herbert 2013). Lack of access to high quality data makes this relationship difficult to study more generally.

### Note 2

For Scanlon, reasons are facts (Scanlon 2014, p. 30, note 20), natural (e.g., that you will enjoy some activity) or normative (e.g., a law’s being unjust or your having reason to go on living; Scanlon 2014, p. 32). These facts are an essential part of a relation to an agent: consideration (or fact) p is a reason for x to do a in circumstances c. ‘Is a reason for’ is a four-place relation, R(p, x, c, a) (pp. 31, 37). For Scanlon there are cases where the relative strength of reasons is derived from the relative amounts of something (such as capabilities), even so he considers it to be a mistake to consider that there must inevitably be a quantitative property which determines the relative strength of reasons (p. 110). ‘The strength of a reason is an essentially comparative notion, understood only in relation to other particular reasons’ (p. 111). When we judge that certain considerations provide conclusive reasons for (or against) certain actions in certain circumstances, justification comes our understanding of a relationship with other rational beings that we have reason to want, specifically, the relationship of seeing them as beings to whom justification is owed (p. 115). Scanlon acknowledges that ‘when we are assessing the justifiability of moral principles we must have reason to appeal to things that individuals have reason to want, and that many of these are things that contribute to well-being intuitively understood.’ However, ‘we cannot delimit the range of considerations that figure in justification by defining the boundaries of well-being’ (Scanlon 1998, p. 140; see Putnam 2008).

### Note 3

Scanlon emphasises the importance of probabilities in determining the degree of effort that we make to control risks. ‘The probability that a form of conduct will cause harm can be relevant not as a factor diminishing the ‘complaint’ of the affected parties ( discounting the harm by the likelihood of their suffering it) but rather as an indicator of the care that the agent has to take to avoid causing harm’. Scanlon states that ‘..the cost of avoiding all behaviour that involves risk of harm would be unacceptable. Our idea of ‘reasonable precautions’ defines the level of care that we think can be demanded: a principle that demanded more than this would be too confining, and could reasonably be rejected on that ground’ (Scanlon 1998, p. 209 & pp. 235–6; see Kumar 2016). Scanlon’s emphasis on reasons allows the inclusion of morally salient considerations such as responsibility and fairness (Scanlon 1998, p. 243). ‘Responsibility of an agent for wrongful conduct, responsibility for creating a situation that gives reason to break a promise, responsibility for engaging in risky conduct that leads to harm and responsibility for misfortune that puts one in need of aid’ (Scanlon 1998, p. 244) are all morally salient considerations. The question that arises is ‘who has responsibility for ‘reasonable precautions’ when it comes to antibiotic resistance in developing countries’, and what is reasonable?
